# Rapidly progressive silicosis previously diagnosed as pulmonary TB in mineworkers

**DOI:** 10.5588/ijtldopen.24.0564

**Published:** 2025-02-01

**Authors:** J. Pyana Kitenge, Q. Said-Hartley, A. Dubbeldam, I. Kabeya Mulaji, P. Kyansa Mangi, P. Musa Obadia, M. Jeebhay, S. Ronsmans, B. Nemery

**Affiliations:** ^1^ KU Leuven Department of Public Health and Primary Care, Centre for Environment and Health, Leuven, Belgium;; ^2^ Département de Santé Publique, Unité de Santé au Travail et Santé Environnementale, Université de Lubumbashi, Lubumbashi, DR Congo;; ^3^ Service de Médecine du travail, Centre Médical Du Centre-Ville (CMDC), Lubumbashi, DR Congo;; ^4^ Department of Radiology, University of Cape Town, Cape Town, South Africa;; ^5^ Department of Radiology, Chest Radiology Unit, University Hospitals Leuven, Belgium;; ^6^ Département de Médecine Interne, Université de Kolwezi, Kolwezi, DR Congo;; ^7^ Département de Santé Publique, Unité de Toxicologie et Environnement, Université de Lubumbashi, DR Congo;; ^8^ Division of Occupational Medicine, University of Cape Town, Cape Town, South Africa.

**Keywords:** chronic respiratory diseases, pneumoconiosis, cobalt-copper mining, Copperbelt-Katanga, Africa-DR Congo

Dear Editor,

In Africa, the high prevalence of pulmonary TB (PTB),^1^ and its many clinical similarities with other chronic respiratory diseases (CRDs),^2,3^ often leads clinicians to treat all CRDs, almost by default, as TB (even in the absence of bacteriological evidence^3,4^). Indeed, in a recent meta-analysis of studies in Africa, nearly 50% of patients with initially presumed TB did not have TB.^5^ Silicosis, with or without TB, is globally the most common occupational lung disease and occurs mainly in mining jobs.^6^ Silicosis is traditionally described as a chronic lung disease that progresses silently over many years from asymptomatic ‘simple silicosis’ towards ‘progressive massive fibrosis’ leading to respiratory insufficiency.^6,7^ Also, (sub)acute presentations with a rapidly fatal outcome may occur in relatively young workers after short but high exposure to crystalline silica.^6–8^ Rapidly progressive or accelerated silicosis has been mainly described among sandblasters^6–8^ (notably denim blasters^7,8^) and workers exposed to dust from engineered stone made of quartz composites.^9^ However, there are few reports of accelerated silicosis among mineworkers (except, possibly, in small-scale mining^10^). Despite extensive mining operations in many African countries, to our knowledge, accelerated silicosis has only been reported in South Africa.^11^ To improve the awareness of clinicians in other African countries, we present two cases that were initially diagnosed as TB but were, in fact, rapidly progressive silicosis. The cases were found in a large hospital in the Katangan Copperbelt, a region of intense historic and current mining activity in the DR Congo. The patients and/or next of kin consented to publication and the submission was approved by the ethical committee of the Université de Lubumbashi, Lubumbashi, DR Congo (UNILU/CEM/015/2023).

Case 1: in June 2023, the chest X-ray of a 40-year-old man showed small round and larger opacities (Figure). He was admitted the previous month for chronic cough and dyspnoea and treated for TB despite a negative GeneXpert test (Cepheid Sunnyvale, CA, USA). The patient was discharged with TB treatment and without known follow-up. However, his occupational history (which had not been previously taken) revealed that the non-smoker patient had worked as an ore-crushing operator in two copper-cobalt mines. He lost his first job after one year due to an unknown respiratory condition but worked for another 3 years at a second mine. Preventive measures were absent in both workplaces. No further clinical details were available in the medical records. The combination of radiological features, the absence of TB, and the high risk of occupational exposure to silica-rich mineral dust led to a diagnosis of silicosis.

**Figure.**
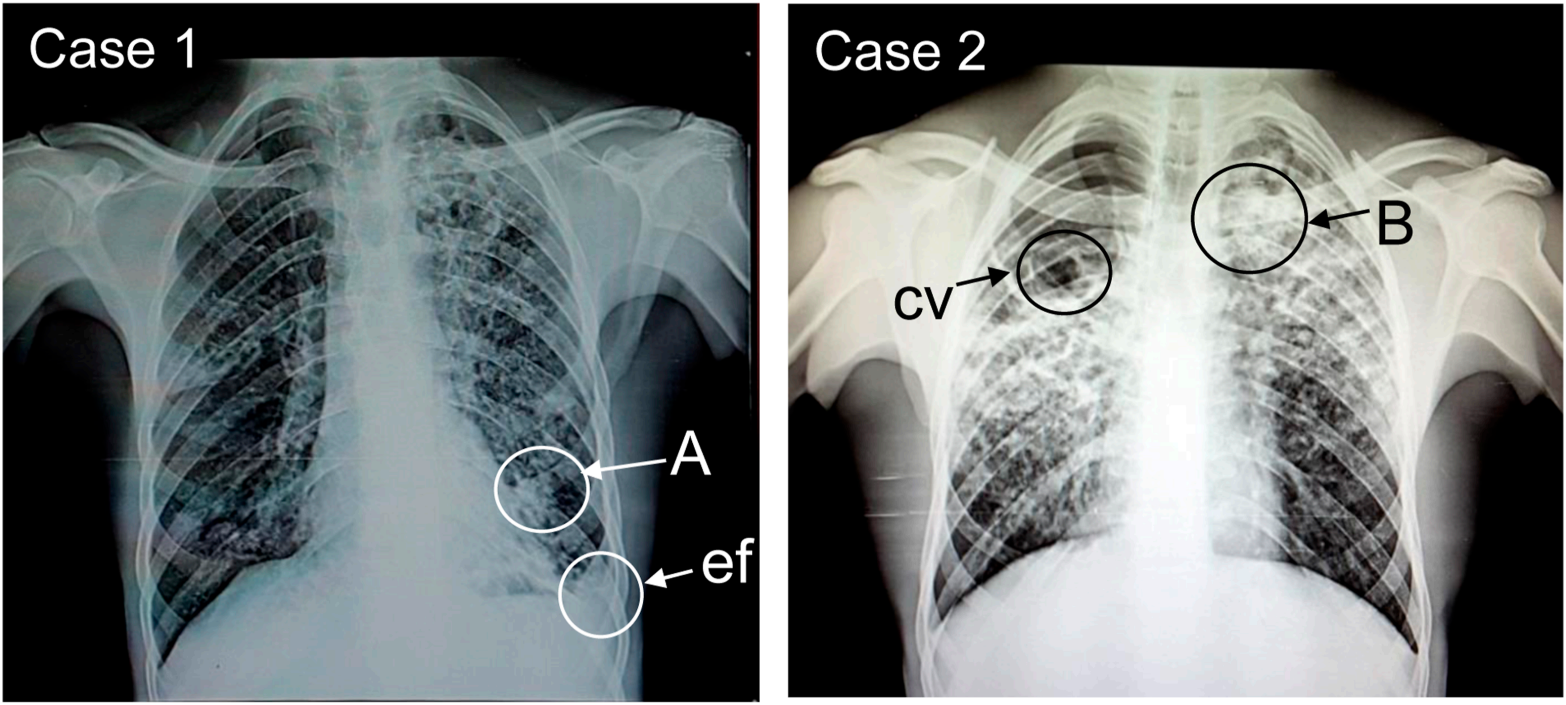
Chest X-rays from two mineworkers, with interpretation according to the ILO International Classification of Radiographs of Pneumoconioses Revised, 2022 edition. Case 1: Radiograph quality Grade 2 (right rotation and inadequate penetration), showing small round opacities, shape and size (q/q) and profusion (2/2), localised in the right middle and lower zones, as well as in the left upper, middle and lower zones. A large opacity (A) next to the left heart border and pleural abnormality: left costophrenic angle obliteration by pleural effusion (ef). In the absence of TB, these radiological features are compatible with silicosis. Case 2: Radiograph quality Grade 2 (left rotation and inadequate penetration) showing diffuse small round opacities, size and shape (r/q), profusion (3/2), localised in the right, middle and lower zones, as well as the left upper, middle and lower zones, and large opacities (B) in the left upper zone and background ground glass opacities; cavity (cv) in right upper zone. These radiological features are suggestive of silico-TB.

Case 2: in June 2023, the chest X-ray of a 23-year-old man showed small, rounded and larger opacities (Figure). He was a non-smoker and had been admitted the previous month for chronic cough, dyspnoea, chest pain and fatigue. He was treated for TB after a positive GeneXpert test. His occupational history (which had not been previously taken) revealed he had been employed for three years as a crushing operator in a copper-cobalt mine. The working conditions suggested high exposure to mineral dust, with little regard for occupational safety and health. The patient was emaciated, but no further clinical details were found in his medical records. The combination of radiological features, the positive TB test, and the risk of high exposure to mineral dust (likely containing crystalline silica) led to a probable diagnosis of silicosis with TB, i.e. silico-TB. The patient died of respiratory failure about one month after admission.

Health workers and public health authorities in low- and middle-income countries should be aware that TB is not the only CRD. We report here on two ore-crushing operators who were treated for PTB, but based on their occupational histories, they were subsequently diagnosed as having silicosis. This misdiagnosis reflects a lack of awareness of silicosis, even in this region of intense mining. In both men, silicosis appeared after relatively short-duration exposure to what we assume to be high levels of dust. No dust measurements are available for these workplaces, but we arranged for X-ray diffraction analysis of a sample of settled dust (obtained in June 2023) from ore-crushing operations in a cobalt-copper mine in Kolwezi, DR Congo. This revealed a composition (by weight) of 37% quartz (unpublished observations). This confirms the potentially high content of crystalline silica when mining and processing cobalt-copper in the area. For Case 1, whose test for TB was negative, the advanced radiologic findings leave little doubt that rapidly progressive silicosis was the underlying condition.^12^ For Case 2, the positive TB test suggests that the disease really was PTB. However, his occupational history as an ore-crushing operator exposed to silica dust, the radiological presentation (small rounded and larger opacities, background ground glass opacities) and rapid disease progression^6,7^ (despite presumably adequate anti-TB treatment) supports a diagnosis of accelerated silicosis rather than just PTB. Although the certainty of our diagnosis of silicosis would have been enhanced through HRCT imaging or histological confirmation,^6–8^ these procedures were not available. However, it is well known that exposure to crystalline silica increases the risk of TB and that silicosis may be complicated by TB.^6,7,12^

CRDs contribute substantially to the burden of disease in low-income countries.^13^ The cases described here are, to our knowledge, the first detailed clinical descriptions of accelerated silicosis in the cobalt-copper mining region of DR Congo. Both had been diagnosed and treated for TB, and their possible work-relatedness had not been considered. Admittedly, these sentinel cases provide only anecdotal evidence of a lack of awareness of occupational diseases. Nevertheless, they give credence to the widely shared impression that clinicians in Africa often tend to consider – and hence, treat – lung disease as being due to TB, even in the absence of bacteriological proof.^3,4^ Given the high prevalence of TB and its amenability to treatment, this is understandable, but it leads to inappropriate treatment. It also leads to an underestimation of the prevalence and incidence of other CRDs, such as chronic obstructive pulmonary disease, lung cancer and pneumoconioses. Moreover, as shown in Case 2, even when a diagnosis of TB is justified, occupational exposure to silica may contribute to its occurrence and severity. In the biennial reports (1970–1995) of Congo’s national mining company, 27 cases of fatal chronic silicosis (11 with TB) were identified.^14^ No information is available about the incidence of pneumoconioses in the industrial, or artisanal mining sector over the past two decades despite the recent mining boom in the ex-Katanga province. In fact, except for South Africa, data about silicosis (and other occupational lung disease) in Sub-Saharan Africa is scarce.^10,15^ Currently, we are performing epidemiological studies to assess the contribution of mining to the burden of respiratory disease in this intensely mined region of DR Congo. The silicosis cases described here were discovered (by JPK) when preparing a case-control study on mining-related interstitial lung diseases. Underdiagnosis of occupational diseases may be explained by the weakness of occupational health in DR Congo. Most health workers in Africa have never received education, let alone training, about occupational disease. Most African countries do not have specialised centres for the prevention and diagnosis of occupational diseases. Nevertheless, as demonstrated here, the primary means of diagnosing occupational diseases is to record the work history of all CRD cases.

We encourage clinicians (including radiologists), as well as all centres dedicated to the diagnosis and treatment of TB, to consider occupational and environmental lung diseases by recording occupational histories. We also call on policymakers to implement environmental and occupational policies, and training programmes to strengthen the prevention of occupational diseases. There is also a need for surveillance programmes for dust-induced diseases, with compensation schemes for individual victims.
